# CD147 Expression in Human Gastric Cancer Is Associated with Tumor Recurrence and Prognosis

**DOI:** 10.1371/journal.pone.0101027

**Published:** 2014-06-30

**Authors:** Dake Chu, Shaojun Zhu, Jipeng Li, Gang ji, Weizhong Wang, Guosheng Wu, Jianyong Zheng

**Affiliations:** 1 State Key Laboratory of Cancer Biology and Xijing Hospital of Digestive Diseases, Xijing Hospital, Fourth Military Medical University, Xi'an, Shaanxi, China; 2 State Key Laboratory of Cancer Biology and Department of Biochemistry and Molecular Biology, Fourth Military Medical University, Xi'an, Shaanxi, China; 3 Department of Pathology, Tangdu Hospital, Fourth Military Medical University. Xi'an, Shaanxi, China; Columbia University, United States of America

## Abstract

CD147 is correlated with tumor aggressiveness in various human malignancies. Here, we investigated CD147 protein expression in 223 patients with gastric cancer by immunohistochemistry and analyzed its association with disease-free and overall survival. CD147 was increased in gastric cancer compared to normal tissues. Additionally, CD147 expression was associated with gastric cancer invasion, metastasis and TNM stage, whereas it was not related to age, sex, differentiation status, tumor site or Lauren classification. Kaplan-Meier analysis confirmed that CD147 was associated with disease-free and overall survival in patients with gastric cancer; i.e., patients with positive CD147 staining tend to have worse disease-free and overall survival. Moreover, Cox's proportional hazards analysis demonstrated that CD147 was an independent marker of disease-free and overall survival for patients with gastric cancer. These results confirm the association of CD147 with gastric cancer invasion and metastasis and prove that CD147 might be an indicator of tumor recurrence and prognosis in gastric cancer.

## Introduction

Gastric cancer is one of the most common human malignancies worldwide [1], [2]. The highest rates of gastric cancer are in Eastern Asian countries. Nearly 42% of male and 19% of female gastric cancer patients throughout the world are found in China, where gastric cancer constitutes the third leading cause of cancer-related death [3], [4]. Surgical resection is still the main treatment for gastric cancer. However, approximately 60% of patients with gastric cancer have locally advanced and metastatic disease at the time of surgery, resulting in a relatively low therapeutic efficacy with surgical resection. The degradation of the extracellular matrix (ECM) is critical for gastric cancer cells to invade into surrounding tissue. Recent studies focusing on the mechanisms underlying gastric invasion suggest that matrix metalloproteinases (MMPs) play a critical role in this process [5].

Extracellular matrix metalloproteinase inducer (EMMPRIN), also known as CD147, is a 57-kilodalton (kDa) transmembrane glycoprotein that belongs to the immunoglobulin (IgG) superfamily [6]. CD147 is composed of 2 extracellular Ig domains, a single transmembrane domain required for counter receptor binding activity which is involved in matrix metalloproteinases (MMPs) induction and a short cytoplasmic domain that interacts with Cav-1 [7], [8]. CD147 is abundantly expressed on the outer surface of many types of tumor tissue compared with normal tissue, and it may facilitate tumor metastasis by modulating cellular substrate and adhesion processes [9]–[11]. CD147 can stimulate adjacent interstitial normal cells to produce MMPs, a group of zinc-dependent proteins that can degrade the extracellular matrix (ECM) [12], [13]. Degradation of the ECM at both the primary tumor site and at the secondary colonization site is critical for tumor invasion and metastasis [12]. Recently, the role of CD147 in tumor invasiveness was confirmed in several human malignancies, including gastric cancer [14]–[16]. Inhibition of CD147 expression by RNA interference reduced tumor cell invasion and tumorigenicity, as well as increased chemosensitivity to paclitaxel [17]. For some tumor types, such as pulmonary adenocarcinoma, seminomas, salivary duct carcinoma, prostate cancer, colorectal cancer, bladder cancer and breast cancer, EMMPRIN expression has been associated with patient prognosis [18]–[22]. In gastric cancer, increased CD147 expression is related to poor overall survival [23]. However, the association of CD147 with gastric cancer recurrence has not been investigated.

In this study, we have analyzed the expression of CD147 in clinical gastric specimens and its association with disease-free survival and overall survival in patients following surgery.

## Materials and Methods

### Clinical gastric cancer specimens

This study was approved by the Ethics Committee of the Fourth Military Medical University. All patients or family members involved provided informed written consent. Briefly, fresh clinical gastric cancer specimens and adjacent normal tissues were collected from 223 patients who underwent surgery between January 2006 and June 2007 in the Xijing Hospital of Digestive Diseases. None of these patients received chemotherapy prior to surgery. In addition, normal tissue samples were taken from 21 patients who underwent surgery for reasons other than malignancy, representing normal control samples. The histomorphology of all tissue specimens was confirmed by the Department of Pathology, Xijing Hospital. Specimens were fixed in 10% formaldehyde and imbedded in paraffin for histological sectioning. Patient clinical information, such as age, sex, differentiation status and TNM stage, was collected and stored in a database. Follow-up information for all of the participants was updated every 3 month using telephone interviews and questionnaires. Disease-free survival is defined as the time elapsed from surgery to the first occurrence of any of the following events: recurrence of gastric cancer; gastric cancer distant metastasis; development of second non-gastric malignancy, excluding basal cell carcinomas of the skin and carcinoma in situ of the cervix; or death from any cause without documentation of a cancer-related event. The diagnosis of recurrence and distant metastasis was based on imaging methods, including endoscopy, ultrasonography, computed tomography, magnetic resonance imaging and position emission tomography, and when possible, cytologic analysis or biopsy. Overall survival is defined as the time elapsed from surgery to death from any cause of patients with gastric cancer.

### Immunohistochemical assays

Fresh tissue specimens were deparaffinized in xylene and dehydrated through a graduated alcohol series. Endogenous peroxidase activity was blocked with 0.5% H_2_O_2_ in methanol for 10 min. For antigen retrieval, sections were microwaved in 0.01 M sodium citrate (pH 6.0). Nonspecific binding was blocked by incubating the sections with 10% normal goat serum in PBS for 1 h at room temperature. Without washing, these sections were incubated with the mouse anti-human CD147 monoclonal antibody (1∶200), which has been validated by previous studies, in PBS at 4°C overnight in a moist box [24], [25]. Then, the sections were incubated with biotinylated rabbit anti-mouse IgG (1∶400) for 1 h at room temperature, and CD147 expression was detected using a streptavidin-peroxidase complex. The brown color indicative of peroxidase activity was developed by incubating the sections with 0.1% 3, 3-diaminobenzidine in PBS containing 0.05% H_2_O_2_ for 5 min at room temperature. Appropriate positive and negative controls were performed in each experiment.

### Evaluation of staining

The tissue specimens were viewed separately by two pathologists without prior knowledge of the clinical or clinicopathological status of the specimens. The staining was evaluated by scanning the entire tissue specimen under low magnification (×40), followed by confirmation under high magnification (×200 and ×400). An immunoreactivity score (IRS) system based on the proportion and intensity of positively stained cancer cells was applied: A: number of positive stained cell ≤5%, scored 0; 6%∼25%, scored 1; 26%∼50%, scored 2; 51%∼75%, scored 3; >75%, scored 4. B: intensity of stain: colorless, scored 0; pale yellow, scored 1; yellow, scored 2; brown, scored 3. The scores from A and B were multiplied, and the staining grade was stratified as negative (-, 0 score), weak (+, 1∼4 score), moderate (++, 5∼8 score) or strong (+++, 9∼12 score). Another pathologist who was blind to the study data also interpreted the stained slides. Specimens were rescored if the difference in the scores from the two pathologists was more than 3 [26], [27].

### Statistical analysis

Associations between CD147 expression and categorical variables were analyzed by Mann Whitney test or Kruskal Wallis test, as appropriate. Survival curves were estimated using the Kaplan-Meier method, and differences in survival distributions were evaluated by the log-rank test. Cox's proportional hazards modeling of factors potentially related to survival was performed to identify factors that might have a significant influence on survival. Differences with a p value of 0.05 or less were considered statistically significant.

## Results

### CD147 Staining in Gastric Cancer

**Figure 1:**
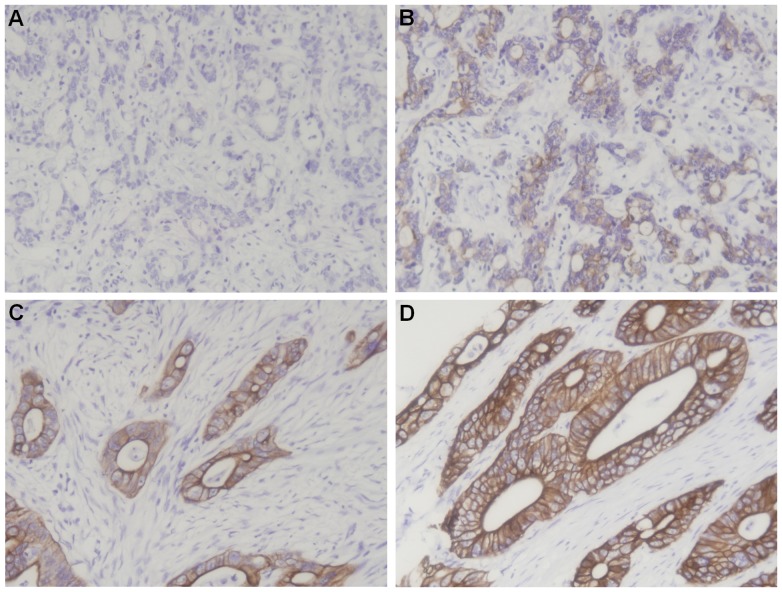
Immunohistochemistry staining pattern of CD147 in gastric cancer (×200). **A** Negative staining (−) of CD147 in gastric cancer; **B** Weak positive staining (+) of CD147 in gastric cancer; **C** Moderate positive staining (++) of CD147 in gastric cancer; **D** Strong positive staining (+++) of CD147 in gastric cancer.

In immunohistochemistry assay results, CD147 staining was mainly localized at the cell membranes and in the cytoplasm of gastric cancer cells. Negative, weak, moderate and strong staining of CD147 evaluated by IRS was showed in Figure 1. The staining pattern of CD147 in gastric cancer was consistent with previous reports [28]. Among the 223 gastric cancer specimens, negative (−) CD147 staining was detected in 90 samples, weak positive (+) CD147 staining was detected in 57 samples, moderate positive (++) CD147 staining was detected in 44 samples and strong positive (+++) CD147 staining was detected in 32 samples. In contrast, among the 223 adjacent normal tissues, negative (−), weak positive (+) and moderate positive (++) CD147 staining was detected in 175 samples, 36 samples and 12 samples, respectively, while no strong positive (+++) staining was detected. The difference in CD147 expression between gastric cancer and adjacent normal tissues was statistically significant (*P*<0.001), indicating that CD147 expression is increased in gastric cancer compared with normal adjacent tissues.

### Association of CD147 Staining with Clinicopathological Characteristics

Based on the IRS scores of immunohistochemical staining, we further analyzed the relationship between CD147 staining and clinicopathological characteristics of patients with gastric cancer (Table 1). CD147 staining was associated with gastric cancer invasion, as stronger positive staining of CD147 was more frequently detected in T3 and T4 tumors compared to T1 and T2 tumors (*P = *0.035). Furthermore, CD147 staining was associated with lymph node metastasis and distant metastasis, as stronger positive staining was more likely to be detected in tumors with lymph node metastasis (*P<*0.001) or distant metastasis (*P<*0.001). CD147 staining was closely related to the TNM stage of gastric cancer, as staining was progressively stronger from grade I to grade IV tumors (*P<*0.001, Table 1). However, CD147 staining was not related to patient sex, age, tumor differentiation, tumor site or Lauren classification (Table 1). These results indicate that CD147 staining is related to gastric cancer invasion and metastasis.

**Table 1:** Relationship between CD147 staining and clinicopathological characteristics.

	n	CD147				*P*
		–	+	++	+++	
**Total**	223	90	57	44	32	
**Gender**						0.798*
Male	120	48	30	24	18	
Female	103	42	27	20	14	
**Age**						0.330*
<60	131	58	29	26	18	
≥60	92	32	28	18	14	
**Differentiation**						0.647†
Poor	70	32	16	12	10	
Moderate	103	40	27	22	14	
Well	50	18	14	10	8	
**Tumor site**						0.056†
Gastroesophageal junction	43	11	14	11	7	
Corpus/Fundus	134	64	29	24	17	
Antrum/Pylorus	46	15	14	9	8	
**Lauren classification**						0.954*
Intestinal	83	33	22	16	12	
Diffuse	140	57	35	28	20	
**Invasion**						0.035*
T1+T2	109	50	28	21	10	
T3+T4	114	40	29	23	22	
**lymph node Metastasis**						*<*0.001*
Negative	97	65	15	11	6	
Positive	126	25	42	33	26	
**Distant Metastasis**						<0.001*
Negative	203	88	52	40	23	
Positive	20	2	5	4	9	
**TNM stage**						<0.001†
I	35	25	4	4	2	
II	62	35	13	8	6	
III	106	23	35	28	15	
IV	20	2	5	4	9	

*P value was estimated by Mann Whitney test.

† *P* value was estimated by Kruskal Wallis test.

### Relationship Between CD147 Expression and Disease-free Survival

**Figure 2:**
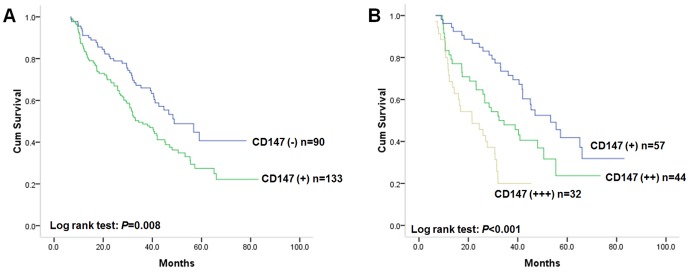
**A** Kaplan-Meier postoperative survival analysis on disease-free survival of primary gastric patients (n = 223) with positive CD147 staining (n = 133) and negative CD147 staining (n = 90). The disease-free survival for patients in negative CD147 staining group was significantly better than that for patients in positive staining group (log rank test: *P* = 0.008); **B** Kaplan-Meier postoperative survival analysis on disease-free survival of primary gastric patients with positive CD147 staining (n = 133). The disease-free survival rate was decreased from weak positive (+, n = 57), moderate positive (++, n = 44) to strong positive (+++, n = 32) CD147 staining group (log-rank test: *P*<0.001).

Because CD147 staining was related to gastric cancer invasion and metastasis, we further analyzed its association with disease-free survival, which is mainly determined by the extent of invasion and metastasis. During the follow-up period, 129 of the 223 patients with gastric cancer presented events. The median disease-free survival time for all patients was 41.8 months (95% CI: 36.2–47.4 months). As there were a relatively small number of samples in the strong positive CD147 staining group due to small sample size, we combined strong, moderate and weak positive CD147 staining into a single group, defined as the positive CD147 staining (+) group. Kaplan-Meier analysis was used to evaluate the disease-free survival of patients with gastric cancer in relation to CD147 expression. Our results indicate that patients with CD147 positive tumors had worse disease-free survival compared with patients with CD147 negative tumors (Figure 2A, log-rank test: *P* = 0.008). The postoperative median disease-free survival time of patients with positive CD147 staining was 34.4 months (95% CI: 27.3–47.5), while that of patients with negative CD147 staining was 48.9 months (95% CI: 35.2–62.6). Further analysis on disease-free survival in subgroups of positive CD147 staining tumors revealed that the survival rate was decreased from weak positive (+), moderate positive (++) to strong positive (+++) CD147 staining group (Figure 2B, log-rank test: *P*<0.001). When the unadjusted hazard ratio (HR) was set to 1.0 (as reference) in CD147 negative (−) staining group, patients with CD147 positive gastric cancer had a 1.63-fold higher risk of relapse compared to those with CD147 negative gastric cancer (95% CI: 1.13–2.36; *P* = 0.009). Regarding clinicopathological characteristics, tumor invasion status (log-rank test: *P* = 0.016), lymph node metastasis (log-rank test: *P*<0.001) and TNM stage (log-rank test: *P*<0.001) were also associated with disease-free survival of patients with gastric cancer. Patients with gastric cancer displaying deep invasion, lymph node metastasis or advanced TNM stage had worse disease-free survival and a higher risk of relapse than those without. However, sex, age, tumor location, differentiation status or Lauren classification had no prognostic value on disease-free survival of patients with gastric cancer. The unadjusted HR is shown in table 2.

**Table 2:** Association between CD147 and clinical factors with disease-free survival.

	Unadjusted HR* (95% CI)	*P*	Adjusted HR† (95% CI)	*P*
**CD147**				
Negative (−)	-		-	
Positive (+)	1.63 (1.13-2.36)	0.009	1.51 (1.05-2.16)	0.026
**Sex**				
Female	-		-	
Male	1.52 (0.81-2.86)	0.112	1.44 (0.86-2.41)	0.171
**Age**				
≤60	-		-	
>60	1.34 (0.92–1.98)	0.131	1.12 (0.76–1.66)	0.567
**TNM stage**				
I	-		-	
II	1.76 (1.12–3.21)	0.021	2.52 (1.51–5.16)	0.005
III	2.26 (1.47–4.68)	0.019	3.14 (1.52–7.28)	0.003
IV	4.35 (1.71–10.22)	<0.001	5.11 (1.92–11.96)	<0.001

*Hazard ratios in univariate models.

† Hazard ratios in multivariable models.

Abbreviations: HR, hazard ratio; 95% CI, 95% confidence interval.

In multivariate analysis, the Cox proportional hazards model was adjusted for gender, age and TNM stage, and CD147 expression was an independent prognostic factor of disease-free survival of patients with gastric cancer. The adjusted HR was 1.00 (as a reference) and 1.51 in CD147 negative and positive patients, respectively (95% CI: 1.05-2.16; *P* = 0.026, Table 2). Thus, CD147 expression in gastric cancer could be an independent predictor of disease-free survival for patients, indicating that patients with positive CD147 staining have a higher risk of relapse than those with negative staining. In addition, TNM stage was also a prognostic factor for disease-free survival in multivariate analysis. (Table 2)

### Relationship Between CD147 Expression and Overall Survival

**Figure 3:**
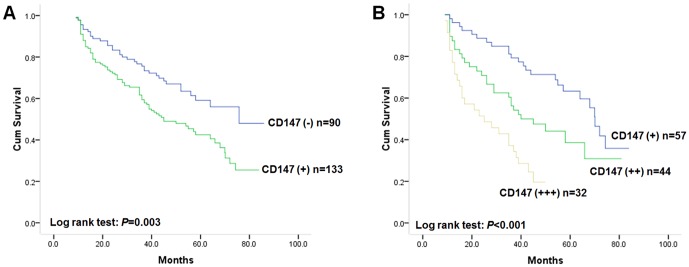
**A** Kaplan-Meier postoperative survival analysis on overall survival of primary gastric patients (n = 223) with positive CD147 staining (n = 133) and negative CD147 staining (n = 90). The overall survival for patients in negative CD147 staining group was significantly better than that for patients in positive staining group (log rank test: *P* = 0.003); **B** Kaplan-Meier postoperative survival analysis on overall survival of primary gastric patients with positive CD147 staining (n = 133). The overall survival rate was decreased from weak positive (+, n = 57), moderate positive (++, n = 44) to strong positive (+++, n = 32) CD147 staining group (log-rank test: *P*<0.001).

Because CD147 staining was related to disease-free survival of gastric cancer, we further analyzed its association with overall survival. The median overall survival time for all patients was 58.0 months (95% CI: 46.7–69.3 months). Kaplan-Meier analysis was used to evaluate the overall survival of patients with gastric cancer indicates that patients with CD147 positive tumors had worse overall survival compared with patients with CD147 negative tumors (Figure 3A, log-rank test: *P* = 0.003). The postoperative median overall survival time of patients with positive CD147 staining was 45.0 months (95% CI: 32.5–57.5), while that of patients with negative CD147 staining could not be estimated due to better overall survival. Subgroup analysis on overall survival revealed that the survival rate was decreased from weak positive (+), moderate positive (++) to strong positive (+++) CD147 staining group (Figure 3B, log-rank test: *P*<0.001). Patients with CD147 positive gastric cancer had a 1.79-fold higher risk of death compared to those with CD147 negative gastric cancer (95% CI: 1.20–2.67; *P* = 0.004, Table 3).

**Table 3:** Association between CD147 and clinical factors with overall survival.

	Unadjusted HR* (95% CI)	*P*	Adjusted HR† (95% CI)	*P*
**CD147**				
Negative (-)	-		-	
Positive (+)	1.79 (1.20–2.67)	0.004	1.59 (1.05–2.40)	0.028
**Sex**				
Female	-		-	
Male	1.09 (0.70–1.67)	0.710	1.03 (0.66–1.60)	0.896
**Age**				
≤60	-		-	
>60	1.04 (0.71–1.50)	0.855	1.02 (0.70–1.49)	0.905
**TNM stage**				
I	-		-	
II	1.91 (1.09–3.36)	0.025	1.96 (1.12–3.48)	0.008
III	2.19 (1.33–3.63)	0.002	2.25 (1.36–3.97)	0.001
IV	3.38 (1.76–6.46)	<0.001	3.96 (1.82–7.39)	<0.001

*Hazard ratios in univariate models.

† Hazard ratios in multivariable models.

Abbreviations: HR, hazard ratio; 95% CI, 95% confidence interval.

In multivariate analysis, the Cox proportional hazards model was adjusted for gender, age and TNM stage, and CD147 expression was an independent prognostic factor of overall survival of patients with gastric cancer. The adjusted HR was 1.59 in CD147 positive patients (95% CI: 1.05–2.40; *P* = 0.028, Table 3). Thus, CD147 expression in gastric cancer could be an independent predictor of overall survival for patients, indicating that patients with positive CD147 staining have a higher risk of death.

## Discussion

Tumor recurrence is a great threat to patients with gastric cancer throughout the world. Even for stage I gastric cancer, approximately 5% to 30% of patients develop recurrent disease and eventually die of metastatic disease. Thus, controlling tumor recurrence is one of the most effective strategies to improve prognosis in gastric cancer management. Chemotherapy reduces tumor recurrence and increases the life expectancy of patients with gastric cancer. However, in addition to its therapeutic effects in tumor cells, chemotherapy will inevitably result in significant clinical toxicity and side effects in normal organs and tissues. Thus, to administer the most effective treatment and minimize side effects, there is an urgent need for more sensitive markers to predict tumor recurrence and prognosis.

In the present study, we investigated CD147 protein expression in 223 cases of clinical gastric cancer and its association with disease-free survival. CD147 protein expression was increased in gastric cancer tissues compared with adjacent normal tissues, and stronger positive staining of CD147 was more frequently detected in gastric cancer with deep invasion, lymph node metastasis, distant metastasis and advanced TNM stage, which was is consistent with previous studies on the relationship between CD147 and human gastric cancer [23]. However, CD147 expression was not correlated with age, gender, differentiation status, tumor site or Lauren classification. Recent studies suggested that RNAi-mediated down-regulation of CD147 decreased cell proliferation, MMP-2 and MMP-9 activities and the invasive potential of SGC7901 cells, which support our results demonstrating the association of CD147 with gastric cancer invasion and metastasis [16]. Taken together, these data suggest that CD147 might be involved in gastric cancer progression.

Because CD147 is associated with gastric cancer invasion and metastasis, which may determine tumor recurrence, we further analyzed its association with disease-free and overall survival. Kaplan-Meier analysis revealed significantly worse disease-free and overall survival for patients with CD147 positive tumors, indicating that CD147 protein level is a marker of recurrence and prognosis for patients with gastric cancer. Cox proportional hazards model adjusted for age, gender and TNM stage showed the same trend as Kaplan-Meier survival analysis, confirming CD147 expression as a marker of poor disease-free and overall survival, independent of known clinical prognostic indicators such as TNM stage. Prolonged disease-free survival in CD147 negative staining group suggested that CD147 might be a potential predictive marker of early recurrence or metastasis in gastric cancers. And overall survival is an essential clinical measurement to evaluate risk of patients. Therefore, CD147 might be a valuable clinical indicator to evaluate the risk of patients with gastric cancer.

The association of CD147 with gastric cancer invasion, metastasis and recurrence may at least be partially due to its effect on MMPs, as MMPs permit cells to actively remodel the extracellular matrix. CD147 is known to promote tumor invasion and metastasis via stimulating MMP synthesis in neighboring fibroblasts [8]. In this perspective, CD147 stimulates the production of MMPs, which could degrade the ECM and facilitate tumor cell invasion and metastasis; thus, promoting tumor recurrence. Recent studies also demonstrated that EMMPRIN not only induces different MMPs but also regulates parameters of the urokinase plasminogen activator (uPA) system and vascular endothelial growth factor (VEGF) [29], [30].

In conclusion, CD147 expression was increased in clinical gastric cancer specimens, and it was associated with tumor invasion and metastasis. Our study also provides the first evidence that CD147 is an independent prognostic factor of both disease-free and overall survival for patients with gastric cancer. These findings also suggest that CD147 might be a potential predictive marker of tumor recurrence and prognosis for patients with gastric cancer.
